# Multiple mutations in lipid-A modification pathway & novel *fosA* variants in colistin-resistant *Klebsiella pneumoniae*

**DOI:** 10.4155/fsoa-2018-0011

**Published:** 2018-07-04

**Authors:** Purva Mathur, Balaji Veeraraghavan, Naveen Kumar Devanga Ragupathi, Francis Yesurajan Inbanathan, Surbhi Khurana, Nidhi Bhardwaj, Subodh Kumar, Sushma Sagar, Amit Gupta

**Affiliations:** 1 Department of Laboratory Medicine, All India Institute of Medical Sciences, New Delhi, 110 029, India; 2 Department of Clinical Microbiology, Christian Medical College, Vellore, 632 004, India

**Keywords:** antimicrobial resistance, colistin resistance, *fosA*, *K. pneumoniae*

## Abstract

**Aim:**

To investigate antimicrobial resistance mechanisms in a cluster of colistin-resistant *Klebsiella pneumoniae*.

**Methods:**

Antimicrobial susceptibility was tested by disk diffusion and broth microdilution. Whole-genome sequencing and genome analysis were performed.

**Results:**

The eight colistin-resistant *K. pneumoniae* isolates belonged to three different clones (ST11, 14 and 231). *The eptA* and *arnT* genes from lipid modification pathway had novel (R157S in *arnT* and Q319R in *eptA*) and rare mutations (V39L, R152H, S260L and A279G in *eptA*). Several substitutions were also identified in *mgrB*, *pmrB*, *phoP* and *phoQ* genes. The *mcr* genes were absent in all isolates. Isolates had variants from existing classes of *fosA* gene.

**Conclusion:**

Complex combination of mutations might have led to colistin resistance, which suggests that continuous surveillance of molecular mechanisms is required.

## Lay abstract

This study focused on identifying antimicrobial resistant mechanisms behind colistin resistance in clinical *Klebsiella pneumoniae* isolates. Novel and rare mutations were identified in the genes involved in the mechanism of colistin resistance. In addition, novel variants of fosfomycin resistance genes were identified in the study isolates. These findings provide an argument for continuous surveillance of colistin resistance.

*Klebsiella pneumoniae* is an important nosocomial pathogen with increasing multi drug resistance capability [1]. At present, there is a limited selection of treatment options for carbapenem-resistant *Enterobacteriaceae* (CRE) infections. There is now a renewed interest in old antimicrobial agents such as polymyxins and fosfomycin.

Data on the activity of fosfomycin against *K. pneumoniae* carbapenemase (KPC)-producing *K. pneumoniae* and New Delhi metallo-β-lactamase (NDM)-1-producing *Enterobacteriaceae* are limited. Use of intravenous fosfomycin monotherapy was proven to effectively control *K. pneumoniae* bacteraemia but can be limited due to its potential resistance development on treatment [2]. Colistin and polymyxin B are known as the most active antimicrobials against CRE [3]. However, in the past few years, there have been sporadic reports of colistin-resistant, CRE cases from various parts of the world including Greece, Israel, South Korea, Singapore and the USA. The exact mechanism(s) of colistin resistance in *Enterobacteriaceae* remain to be unveiled.

Resistance to colistin is mediated mainly via alteration in the lipopolysaccharides of bacterial outer membrane. The alterations include mutations in lipid A modifying genes. The most commonly reported mutations were in the *mgrB* gene and therefore were not transferable through horizontal gene transfer [4]. However, in 2015, the first plasmid-mediated colistin resistance gene (*mcr-1*) was reported [5], which belongs to the phosphoethanolamine transferase enzyme family (*Ept A*). The *mcr-1* was identified in *Escherichia coli* from human patients and animals in China. In 2016, another study reported the mobilizable colistin resistance gene, *mcr*-2 from porcine and bovine *E. coli* isolates in Belgium [6].

Various fosfomycin-modifying enzymes have been identified that act by inactivating the drug. FosA, FosB and FosX are the commonly reported metalloenzymes, while FomA and FomB are kinases. FosA was initially found from a plasmid in *Serratia marcescens* associated with TN2921 transposon [7], while other related FosA type enzymes being reported are FosA3, FosA4, FosA5 and FosC2 [8].

In this study, we performed whole-genome shotgun sequencing of a cluster of colistin-resistant *K. pneumoniae* isolates from North India to identify the molecular mechanism.

## Materials & methods

### Isolates studied

A cluster of eight *K. pneumoniae* isolates from clinical samples (blood, bronchoalveolar lavage and urine) resistant to colistin were chosen for complete molecular characterization, using PCR and next-generation sequencing.

### Antimicrobial susceptibility testing

#### Disc diffusion

All eight isolates were screened for antimicrobial susceptibility by Kirby–Bauer method using amikacin (30 μg), chloramphenicol (30 μg), tetracycline (30 μg), gentamicin (10 μg), ciprofloxacin (5 μg), cefotaxime (30 μg), cefoxitin (30 μg), ceftazidime (30 μg), cefpodoxime (10 μg), piperacilllin-tazobactam (100/10 μg), cefoperazone-sulbactam (75/30), netilmicin (30 μg), imipenem (10 μg), meropenem (10 μg) and tigecycline (15 μg), according to guidelines suggested by CLSI M100-S25, 2015. Quality control strains used were *E. coli* ATCC 25922 for all antibiotics concurrently in all the batches. Tigecycline results were interpreted according to the US FDA criteria.

#### MIC testing

MIC values were determined for meropenem and colistin by broth microdilution method. E-test was performed for fosfomycin MIC using strips with glucose 6-phosphate (bioMérieux, Marcy-l'Etoile, France). *E. coli* ATCC 25922 and Pseudomonas aeruginosa ATCC 27853 were used as quality control strains for MIC determination of meropenem, fosfomycin and colistin with the expected ranges of 0.008–0.06 μg/ml, 0.5–2 μg/ml and 0.25–2 μg/ml for *E. coli* and 0.12–1 μg/ml, 2–8 μg/ml and 0.5–4 μg/ml for *P. aeruginosa*, respectively. The interpretive criterion provided by CLSI 2015 for susceptible, intermediate and resistant strains were ≤4, 8 and ≥16 μg/ml for meropenem, and ≤64, 128 and ≥256 for fosfomycin, respectively. As per EUCAST 2015, isolates with ≤2 and >2 μg/ml MIC were recorded as susceptible and resistant for colistin, respectively.

### PCR for screening of plasmid-mediated colistin resistance genes

Isolation of total DNA was performed using QIAamp DNA mini kit as per manufacturer's instructions (Qiagen, Hilden, Germany). The amplification of colistin resistance genes *mcr-1* and *mcr-2* [5] & [6] was performed using Veriti Thermal cycler (Applied Biosystems, CA, USA).

### Next-generation sequencing

Isolates were further analyzed by whole genome sequencing. Genomic DNA was extracted with QIAamp DNA mini kit (Qiagen, Hilden, Germany). Whole genome sequencing was performed using Ion Torrent (PGM) sequencer with 400-bp read chemistry (Life Technologies, CA, USA) according to manufacturer's instructions. The data were assembled *de novo* using AssemblerSPAdes version 5.0.0.0 embedded in Torrent suite server version 5.0.3. The sequence annotation was performed in PATRIC, the bacterial bioinformatics database and analysis resource [9], Rapid Annotation using Subsystem Technology (RAST) pipeline [10] and NCBI Prokaryotic Genome Automatic Annotation Pipeline. Downstream analysis was done in the Center for Genomic Epidemiology server (www.cbs.dtu.dk/services), RAST and PATRIC. The sequence data were used to perform relativeness analysis by eBURST V3, and UPGMA dendogram was generated using MEGA 7. This Whole Genome Shotgun project has been deposited at DDBJ/ENA/GenBank.

### Statistical analysis

Genome coverage and other parameters were calculated using SPSS 16.0 and Microsoft Excel 2007 (IL, USA).

## Results

### Antimicrobial susceptibility

The resistance pattern for the colistin-resistant *K. pneumoniae* isolates (n = 8) were as given in Table 1. All eight isolates were resistant to cefpodoxime, cefotaxime, ceftazidime, cefoxitin, ciprofloxacin, gentamicin, amikacin, netilmicin and cefoperazone/sulbactam. Isolates except PM5186 were resistant to meropenem and all eight were resistant to colistin by broth microdilution. MICs for colistin ranged from 4–16 μg/ml. Isolate PM716 was resistant to fosfomycin while the remaining were either intermediate or susceptible (Table 1).

**Table 1.** **Phenotypic susceptibility testing and polymerase chain reaction data of colistin-resistant *Klebsiella pneumoniae.***

**Isolate ID**	**CPD-TAX-CAZ- FOX-CIP-GEN-AMK-NET-CFS**	**PTZ**	**CHL**	**TET**	**TIG**	**IMI**	**MEM**	**Meropenem MIC**	**Colistin MIC**	**Fosfomycin MIC**
PM1168	R	R	R	S	S	R	R	32	16	64

PM565	R	R	S	R	R	R	R	32	8	64

PM716	R	R	S	S	S	R	R	64	4	1024

PM1134	R	R	S	S	S	R	R	128	4	128

PM1842	R	R	S	S	S	R	R	16	8	64

PM1995	R	R	S	S	S	S	S	8	8	128

PM138	R	R	S	R	R	R	R	8	8	64

AMK: Amikacin; CAZ: Ceftazidime; CHL: Chloramphenicol; CIP: Ciprofloxacin; CFS: Cefoperazone/sulbactam; CPD: Cefpodoxime; FOX: Cefoxitin; GEN: Gentamycin; IMI: Imipenem; MIC: Minimum inhibitory concentration; MEM: Meropenem; NET: Netilmicin; PTZ: Piperacillin/tazobacam; TAX: Cefotaxime; TET: Tetracycline; TIG: Tigecycline.

### Genome analysis

Raw read assembly of the genome data presented 105–160 contigs (≥ 500 bp). The genome coverage of these isolates were about 32x–51x. The coding sequences (CDSs) of the genomes range from 5859 to 6744, rRNAs from 11 to 14, tRNAs from 64 to 73. The annotation revealed multiple antimicrobial resistance genes ranging from 26 to 38 from ARDB database, and 75–97 from CARD database (www.patricbrc.org). Similarly, for virulence genes, the virulence factor database (VFDB) and Victors database revealed the presence of 81–106 and 177–188 genes, respectively (www.patricbrc.org) (Table 2). Whole genome sequencing of all eight isolates were deposited in Genbank/DDBJ under the accession numbers as follows: PM565 - MNPB00000000; PM1842 - MNPC00000000; PM1995 - MNPD00000000; PM138 - MNPG00000000; PM716 - MNPH00000000; PM1134 - MNPF00000000; PM5186 - MNPE00000000 and PM1168 - MNPA00000000.

**Table 2.** **Whole genome characteristics of colistin-resistant *Klebsiella pneumoniae.***

**Parameters/isolates**	**PM1168**	**PM565**	**PM1842**	**PM1995**	**PM716**	**PM1134**	**PM5186**	**PM138**
Genome Size (bp)	5,554,499	5,650,616	5,577,703	5,583,954	5,727,677	5,735,750	5,440,451	5,774,696

Genome coverage	32x	40x	43x	51x	43x	40x	41x	38x

Number of contigs (≥500 bp)	119	127	110	105	108	132	111	160

CDS	5907	5945	5859	6056	6415	6615	6194	6744

rRNA	11	14	14	13	13	12	12	13

tRNA	68	69	70	70	69	64	73	66

ARDB	35	27	26	29	38	36	36	34

CARD	84	77	75	82	96	97	91	89

VFDB	82	81	82	89	69	67	85	106

Victors	180	177	177	184	187	185	187	188

CDS: Coding seqences; VFDB: Virulence factor database.

**Figure 1.**
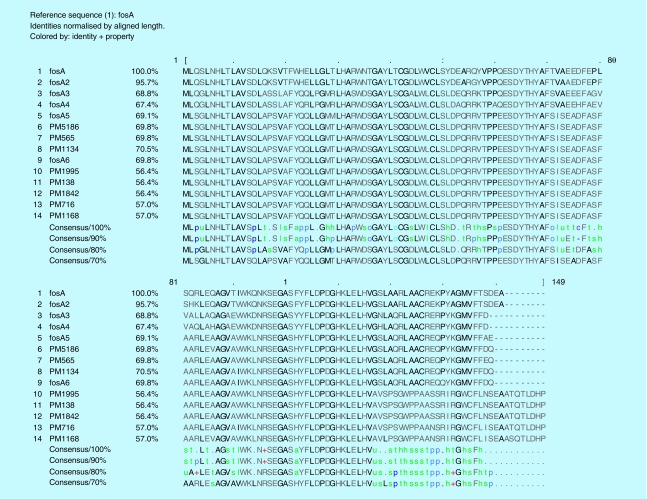
**Clustal alignment depicting amino acid differences among the FosA proteins observed in the study isolates and known variants of FosA.**

The sequence type of the isolates were found to be ST11 for PM565, PM1842, PM1995, PM138, ST14 for PM716, PM1134, and ST231 for PM5186 and PM1168 as analysed by MLST 1.8 tool (https://cge.cbs.dtu.dk//services/MLST/). ResFinder 2.1 (www.cbs.dtu.dk/services) returned multiple antimicrobial resistance genes for most of the antibiotic classes (Table 3). Interestingly fosfomycin, fluoroquinolone, aminoglycoside and β-lactam resistant determinants were found in all eight isolates. The *fosA* genes observed in these isolates were different from the existing six variants and reported for the first time in this study (Figure 1). However, plasmid mediated colistin resistance determinants *mcr-1* and *mcr-2* were not found in any of the isolates.

**Table 3.** **Antimicrobial resistance genes and plasmid profiles of colistin-resistant *Klebsiella pneumoniae.***

**Isolate ID**	**Aminoglycosides**	**Beta lactams**	**Fluoroquinolones**	**Fosfomycin**	**Macrolide**	**Phenicol**	**Rifampicin**	**Sulphonamide**	**Trimethoprim**	**Plasmids**
PM565	*aacA4, strA, strB, rmtf, aac(6′)Ib-cr*	*blaOXA-232, blaTEM-1B, blaCTX-M-15, blaSHV- 11*	*qnrB1*	*fosA*	–	*–*	*ARR- 2*	*sul2*	–	ColKP3, IncR, IncFII(K), IncHI1B, IncFIB(pQil)

PM1842	*aacA4, strA, strB, rmtf, aac(6′)Ib-cr*	*blaOXA-232,blaTEM- 1B,blaCTX-M-15, blaSHV- 11*	*qnrB1*	*fosA*	*–*	*–*	*ARR- 2*	*sul2*	–	ColKP3, IncR, IncFII(K), IncHI1B, IncFIB(pQil)

PM1995	*aacA4, strA, strB, rmtf, aac(6′)Ib-cr*	*blaTEM- 1B, blaCTXM-15, blaLEN12*	*qnrB1*	*fosA*	*–*	*–*	*ARR- 2*	*sul2*	–	IncR, IncFII(K), IncHI1B, IncFIB(pQil)

PM138	*aacA4, strA, strB, rmtf, aac(6′)Ib-cr*	*blaOXA-232, blaTEM-1B, blaCTX-M-15, blaSHV-11*	*qnrB1*	*fosA*	*–*	*–*	*ARR-2*	*sul2*	–	ColKP3, IncR, IncFII(K), IncHI1B, IncFIB(pQil)

PM716	*aadA2, aadA1, aacA4, armA, aph(3′)-Via, aac(6′)Ib-cr*	*blaOXA-232, blaTEM-1B, blaOXA-9, blaOXA-1, blaSHV-28, blaNDM-1, blaCTX-M-15*	*qnrS1, oqxA, oqxB*	*fosA*	*msr(E), mph(E)*	*catB3*	*–*	*sul1*	*dfrA12, dfrA1*	IncHI1B, IncFIB(Mar), IncFIB(pQil), IncFII(K), ColKP3

PM1134	*aadA2, aadA1, aacA4, armA, aph(3′)-Via, aac(6′)Ib-cr*	*blaOXA-181, blaTEM-1B, blaOXA-9, blaOXA-1, blaSHV-28, blaNDM-1, blaCTX-M-15*	*qnrS1, oqxA, oqxB*	*fosA*	*msr(E), mph(E)*	*catB3*	*–*	*sul1*	*dfrA12, dfrA1*	IncHI1B, IncFIB(Mar), IncFIB(pQil), IncFII(K), ColKP3

PM5186	*aacA4, rmtf, aadA2, aac(6′)Ib-cr*	*blaTEM-1B, blaSHV-1, blaCTX-M-15*	*oqxA, oqxB*	*fosA*	*mph(A), erm(B)*	*catA1*	*ARR-2*	*sul1*	*dfrA12*	IncFIB(pQil), IncFII(K), IncFIA

PM1168	*aadA2, rmtf, aacA4, aac(6′)Ib-cr*	*blaOXA- 232, blaTEM- 1B, blaSHV-12, blaCTXM-15*	*qnrS1, oqxA, oqxB*	*fosA*	*mph(A), erm(B)*	*catA1*	*ARR- 2*	*sul1*	*dfrA12*	IncFIB(pQil), IncFII(K), IncFIA

### Plasmid analysis

Plasmids screening was performed using PlasmidFinder 1.3 (https://cge.cbs.dtu.dk//services/PlasmidFinder/). On analysis of plasmids using PlasmidFinder 1.3 (www.cbs.dtu.dk/services), IncFII(K) and IncFIB(pQil) were found in all isolates in addition to few other plasmids (Table 3).

### Mutational analysis

Multiple mutations were observed in the genes responsible for lipid A modification and Ara-4 N pathway in *K. pneumoniae* isolates (Table 4). Interestingly, novel (R157S in *arnT* & Q319R in *eptA*) and rare mutations (V39L, R152H, S260L, A279G in *eptA*) were observed in the isolates studied. R157S in *arnT* was observed in all the isolates, whereas Q319R in *eptA* was observed in PM565, PM1995, PM138 and PM1842. It is important to note that there were deletions of three amino acids LLG at 521, 522 and 523 (Table 4). PM1168 and PM5186 had mutations in *mgrB* gene, V1A and L24H, respectively.

**Table 4.** **Cumulative results of various mutations (amino acid) found upon whole genome sequencing analysis of colistin-resistant *Klebsiella pneumoniae.***

**Genes**		**Lipid A modifications**								**Lipid A-Ara4N pathway (polymyxin resistance)**						

**Isolate**	**Sequence types**	***MgrB***	***PagP***	***PhoQ***	***PhoP***	***PmrA***	***PmrB***	***EptA***	***EptB***	***ArnA_ DH/FT***	***ArnB***	***ArnC***	***ArnT***	***PmrJ***	***PmrL***	***PhoB***
PM565	ST11	None	F170I	D150G	R114A	None	D150H R256G L344P	C27F V39L A279G Q319R	None	I260L N442K	None	S30T	Q156H R157S R372K	W52L V53I I94L I300V	None	None

PM716	ST14	None	F170I	D150G	R114A	None	T157P A246T L344P	V42L S260L	None	I260L N442K	G47D A112D I126V	S30T	Q156H R158S R372K	S164P	None	None

PM1134	ST14	None	F170I	D150G	R114A	None	T157P A246T L344P	V39L S257L	None	I260L N442K	G47D A112D I126V	S19T	Q156H R157S R372K	S164P	None	None

PM1168	ST231	V1A	None	D150G	R114A	None	L344P	V39L, R152H D477N	None	I260L N442K	A112D D285E	S30T	Q156H R157S I474N	NONE	None	None

PM1995	ST11	None	F190I	D150G	R114A	None	D150H R256G L344P	C27F V39L Q319R	None	I260L N442K	None	S30T	Q156H R157S R372K	I94L I300V	None	None

PM138	ST11	None	F189I	D150G	R114A	None	D150H R256G L344P	C27F V39L Q319R	None	I260L N442K	None	S19T	Q156H R157S R372K	I94L I300V	None	None

PM5186	ST231	L24H	None	D150G	R114A	None	L344P	V39L R152H D477N	None	I260L N442K	A112D D285E	S30T	Q156H R157S I474N	NONE	NONE	None

PM1842	ST11	None	F170I	D150G	R114A	None	D150H R256G L344P	C27F V39L Q319R A279G 521,522, 523: DEL LLG	None	I260L N442K	None	S30T	Q156H, R157S R372K	I94L I300V	None	None

## Discussion

Currently, there are increasing reports of CRE which results in less choice of antimicrobials for therapy. Fosfomycin is gaining interest for the treatment of carbapenem-resistant *K. pneumoniae* [2]. In this scenario, resistance to fosfomycin is an alarming threat to those treating infections by *Enterobacteriaceae*, especially nosocomial pathogens.

**Figure 2.**
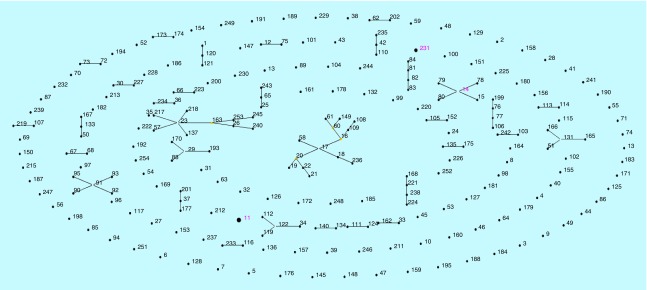
**Comparative eBURST of colistin-resistant *Klebsiella pneumoniae* isolates based on MLST.**

**Figure 3.**
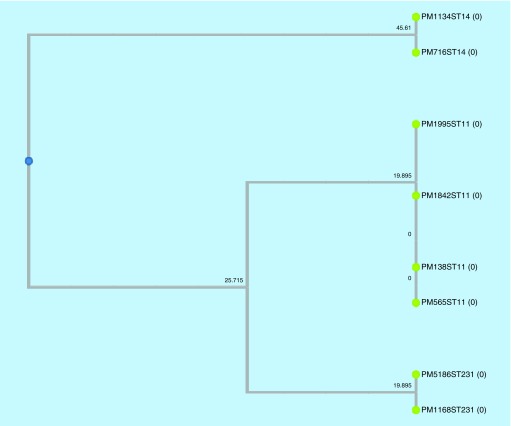
**Dendrogram of colistin-resistant *Klebsiella pneumoniae* isolates to show the clonal similarity using dendroUPGMA.**

The eight selected colistin-resistant *K. pneumoniae* isolates were observed to be of three different clonal types (ST11, ST14 and ST231) as observed by eBURST analysis and UPGMA dendogram (Figures 2 & 3). These were the commonly reported sequence types previously reported from India [11]. Also, among the seven meropenem-resistant isolates, *bla*_OXA_ producers; *bla*_OXA-232_ (n = 5) and *bla*_OXA181_ (n = 1) were commonly seen followed by *bla*_NDM-1_ (n = 2).

Polymyxins are known to serve as drug of choice for carbapenem-resistant *K. pneumoniae* either alone or in combination. Polymyxin acts like cationic detergents and disrupts the cytoplasmic membrane by attacking phosphate groups of membrane phospholipid; this ultimately leads to leakage of cytoplasmic contents and death of cell [12]. In this regard, lipid A modification genes were largely known to be involved in chromosomal-mediated colistin resistance. Among the seven genes which are known to be involved in the lipid A modifications (*paqP*, *pmrA*, *pmrB*, *phoP*, *phoQ*, *eptA* and *eptB*), only four genes (*pmrA*, *pmrB*, *phoP* and *phoQ*) were extensively discussed in the literature. In *K. pneumoniae*, mutations including G53C, E35A in *pmrA* [13]; S85R, T140P, T157P, S205P [13], T157P [14], T157P and S208N with deletion of three nucleotide at 14 and 209 in *pmrB* [15]; L26Q in *phoP* [13]; S174N and L384Q in *phoQ* [15] were previously reported.

In this study, mutations were observed in ten genes (*paqP*, *pmrB*, *phoP*, *phoQ* and *eptA* of lipid A modifications and *arnA_DH/FT*, *arnB*, *arnC*, *arnT* and *pmrJ* of lipid A-Ara4N pathway) which includes novel (R157S in *arnT* & Q319R in *eptA*) and rare mutations (V39L, R152H, S260L and A279G in *eptA*) which might be conferring for colistin resistance. Recently, in one of our studies, we have observed novel mutations in *eptA* gene of lipid A modification pathway and *arnT* gene of lipid A-Ara4N pathway among cluster of isolates from South India [4]. Also, two study isolates (PM1168 and PM5186) exhibited mutations in *mgrB* gene, the most commonly reported genetic determinant for colistin resistance. L24H observed in PM5186 was previously reported by Cannatelli *et al*. [16], whereas V1A (GTG - GCC) was not previously reported. However, no major change was observed in colistin MIC levels for the isolates with and without mutation in *mgrB*. The role of observed mutations in colistin resistance development should be further analyzed with confirmatory tests.

It is also worth noting that there were deletions of LLG aminoacids in *eptA* gene in one isolate (PM1842). The *arnT* gene belonging to L-Ara4N moiety and *eptA* were known to be responsible for attachment of modified arabinose to lipid A 4′-phosphate group. This reduces bacterial susceptibility towards polymyxin and cationic antimicrobial peptides [17].

Interestingly, plasmid-mediated colistin resistance genes *mcr-1* and *mcr-2* were not seen in these isolates. In spite of their absence, the isolates were resistant to colistin (with MICs 8 and 16 μg/ml) indicating the clinical importance of chromosomal mutations in the lipid A modification pathway.

**Figure 4.**
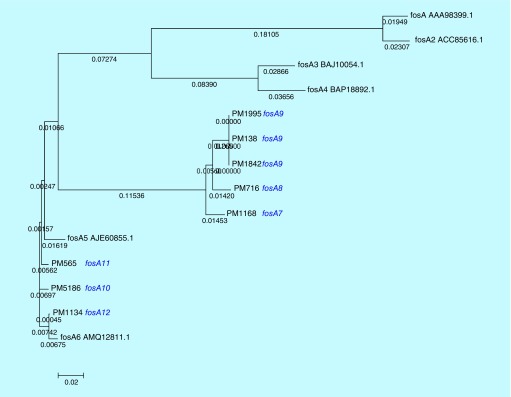
**Dendrogram of novel *fosA* variants in colistin-resistant - *Klebsiella pneumoniae* isolates.**

In addition, intravenous fosfomycin had been proposed as a treatment option for systemic infections by resistant *K. pneumoniae* [18]. However, resistance may develop to fosfomycin during treatment. Resistance to fosfomycin involves various mechanisms, majorly chromosomal-mediated and plasmid-mediated. Transferable plasmids with fosfomycin-resistant determinants result in accelerated dissemination of fosfomycin resistance. Also, *fosA* and *fosB* were reported to be responsible for plasmid-mediated resistance, whereas *fosX* was cited to be responsible for chromosomal-mediated resistance [18]. The *fosA* gene encodes a glutathione S-transferase and *fosB* encodes an L-cysteine thiol transferase, while *fosX* encodes an epoxide hydrolase [19]. Among these *fosA* seems to be widely reported in *K. pneumoniae* isolates [20–22]. To date, six variants of *fosA* have been reported worldwide which includes *fosA* (NC_011617.1), *fosA2* (ACC85616.1), *fosA3* (NC_019073.1), *fosA4* (WP_034169466.1), *fosA5* (NC_022374.1) and *fosA6* (AMQ12811.1). However, to the best of our knowledge, reports are lacking at variant level identification of *fosA* from India. In this study we observed clusters of *K. pneumoniae* isolates with novel *fosA* variants. The variant numbers (PM1168 – *fosA7*; PM716 – *fosA8*; PM138, PM1995, PM1842 – *fosA9*; PM5186 – *fosA10*; PM565 – *fosA11* and PM1134 – *fosA12*) were assigned based on the phylogenetic variation of *fosA* genes (Figure 4). The isolate with *a fosA8* gene had a high MIC of 1024 μg/ml for fosfomycin. However, all other variants of *fosA* genes reported in this study were noted to be either susceptible or moderately susceptible to fosfomycin. In addition, eight more *K. pneumoniae* isolates were screened for *fosA* genes, where all eight were positive for the gene but phenotypically susceptible to fosfomycin. Further studies are required to understand the mechanisms behind fosfomycin resistance and the non-functional variants.

## Conclusion

Overall the study reports novel and rare mutations in the *arnT* gene of the Ara-4 N pathway and the *eptA* gene of lipid A modifications. The complex combination of such mutations leads to high MIC levels for colistin. The result of the study provide an argument for continuous surveillance of the molecular mechanism behind the colistin resistance.

## Future perspective

Most recently, colistin resistance is rapidly increasing among *K. pneumoniae*. The major mechanism reported for colistin resistance is mutations in lipid A modification genes, in which several novel mutations are being reported. Functional validation of such mutations might reveal the level of resistance with each mutation. Plasmid-mediated colistin resistance is seen predominantly in animals, while chromosomal-mediated resistance is higher in humans. It is important to better understand resistance mechanisms – either chromosomal- or plasmid-mediated – and the trend of plasmid-mediated resistance will help us to delineate transmission dynamics of animal to human spread. This information will facilitate the appropriate containment of colistin-resistant pathogen infections.

**Summary points**

- To the best of our knowledge, variants of *fos A* from India have not yet been characterized. The study reports novel variants of *fos A* genes at amino acid level from colistin-resistant *Klebsiella pneumoniae*. The variant numbers (PM1168 – *fosA7*; PM716 – *fosA8*; PM138, PM1995, PM1842 – *fosA9*; PM5186 – *fosA10* and PM565 – *fosA11*) were assigned based on the phylogenetic variation of *fosA* genes.
- The study also reports novel mutations in *arnT* gene of Ara-4 N pathway and rare mutations in *eptA* gene of lipid A modification pathway involved in contributing to colistin resistance.
- The plasmid-mediated colistin resistance genes *mcr-1* and *mcr-2* were absent in all eight *K. pneumoniae* isolates.
- *The bla*_OXA-232_ was seen in most of the isolates, conferring resistance to carbapenem.
- IncFII(K) and IncFIB(pQil) plasmids were seen predominantly in all isolates.
- The common sequence types observed from this study were ST-11 followed by ST-14 and ST-231.
